# Implementing social interventions in primary care in Canada: A qualitative exploration of lessons learned from leaders in the field

**DOI:** 10.1371/journal.pone.0281112

**Published:** 2023-02-16

**Authors:** Gary Bloch, Linda Rozmovits

**Affiliations:** 1 St. Michael’s Department of Family and Community Medicine, Unity Health Toronto, Toronto, Ontario, Canada; 2 Inner City Health Associates, Toronto, Ontario, Canada; 3 Department of Family and Community Medicine, Faculty of Medicine, University of Toronto, Toronto, Ontario, Canada; 4 Independent Qualitative Health Researcher, Montreal, Quebec, Canada; Georgia Southern University, UNITED STATES

## Abstract

**Purpose:**

Primary health care providers and practices are increasingly instituting direct interventions into social determinants of health and health inequities, but experiences of the leaders in these initiatives remain largely unexamined.

**Methods:**

Sixteen semi-structured interviews with Canadian primary care leaders in developing and implementing social interventions were conducted to assess barriers, keys to success, and lessons learned from their work.

**Results:**

Participants focused on practical approaches to establishing and maintaining social intervention programs and our analysis pointed to six major themes. A deep understanding of community needs, through data and client stories, forms a foundation for program development. Improving access to care is essential to ensuring programs reach those most marginalized. Client care spaces must be made safe as a first step to engagement. Intervention programs are strengthened by the involvement of patients, community members, health team staff, and partner agencies in their design. The impact and sustainability of these programs is enhanced by implementation partnerships with community members, community organizations, health team members, and government. Health providers and teams are more likely to assimilate simple, practical tools into practice. Finally, institutional change is key to establishing successful programs.

**Conclusion:**

Creativity, persistence, partnership, a deep understanding of community and individual social needs, and a willingness to overcome barriers underlie the implementation of successful social intervention programs in primary health care settings.

## Introduction

While family physicians and other health care providers have long recognized that social circumstances are the most powerful determinants of health [1], historically many have felt unsure how to directly mitigate the associated risks [2, 3]. A growing number of primary care providers are seeking to address this situation by developing approaches to the social risks to health. These approaches include the use of social risk screening tools, income security interventions, medical-legal partnerships, social prescribing initiatives, and employment assistance interventions [4–9]. The field of social interventions is now broad enough that we can begin to identify lessons learned and approaches to their development and implementation.

Primary health care has been described as sitting on a boundary between traditional, narrowly focused, biomedical approaches to care and a holistic perspective on health that necessitates action on social context to improve outcomes [10]. As such, primary health care is well placed to lead the reorientation of health systems to focus on addressing social determinants of health [11, 12]. Family physicians are generally sympathetic to the impact of social determinants on health outcomes, but many do not feel it is their role to take direct political or social action to address that impact [13]. Barriers to addressing social risks to health include provider prejudice and resistance to innovation, limited resources, fear of addressing power dynamics and structural inequities within teams, an absence of accepted standards of care, and a lack of knowledge of specific interventions [14–16]. While primary care providers express concern about their ability to include social interventions in an increasingly busy scope of practice, access to social interventions has been shown to reduce burnout and improve primary care provider job satisfaction [17, 18].

The literature on social interventions in primary care has focused largely on describing initiatives, evaluating their implementation and, to a lesser extent, on outcomes for primary care providers and their patients [4, 19]. Prior qualitative studies have explored attitudes and general approaches to health inequities among primary care providers and managers [13, 20], and the impact of specific interventions on health team structures and processes [21]. In this study, we seek to describe specific approaches, enablers and barriers to the success and sustainability of social interventions, through an examination of the experiences of primary health care providers, thought leaders and health team managers who have implemented initiatives across Canada.

## Methods

This qualitative descriptive study employed semi-structured, in-depth interviews with sixteen Canadian primary care thought leaders, health practitioners and health team managers who have designed and implemented interventions into the social determinants of health. Purposive (non-probability) sampling, which seeks to maximize theoretical return by allowing for variation within a focused field of inquiry, was used. Potential subjects were identified as leaders in the development and implementation of social intervention initiatives, through professional networks, targeted internet searches, literature review, and snowball referrals. A written consent document was emailed to each participant and verbal consent obtained before the start of their interview. Interviews were conducted by the qualitative researcher (LR), transcribed, and checked against sound files for accuracy.

Interviews explored:

- Drivers of efforts to increase primary care responsiveness to SDOH
- Experiences of developing and implementing initiatives
- Relationships that enabled or hindered this work
- Funding and timing of initiatives
- Introduction of initiatives to colleagues and change management
- Suggestions for building a primary care culture open to these interventions

A coding framework was developed in discussion with the study team that incorporated both anticipated and emergent themes (anticipated themes were identified by the investigators on the basis of the literature and their knowledge of the field). Initial organization of the data using open coding was undertaken by the qualitative researcher. Discussion with the study team then informed development of axial codes to map out the relationships between categories. Selective coding articulated a thematically organized narrative account of the data. The constant comparative method was used to test the integrity of the coding framework and included searches for disconfirming evidence.

A qualitative descriptive approach [22] informed the analysis. This was most appropriate given the applied health services research context and the aim of producing a detailed account of a complex change process as perceived by participants in that process. Data analysis was carried out by the two authors, one of whom (LR) is an experienced critical qualitative health researcher, and the other (GB) an academic family physician and expert in social interventions in primary care. HyperResearch software was used to facilitate data coding and management.

Research ethics board approval was obtained through the St. Michael’s Hospital REB.

## Findings

Invitations to participate were issued to twenty-eight individuals in seven Canadian provinces. Sixteen participated, eleven did not respond and one declined. Participants were interviewed between February and September 2019. Interviews lasted 26–55 (average 38) minutes. Participant characteristics are outlined in Table 1.

**Table 1:** Participant characteristics.

Characteristics	N (%)
Professional Designation (some participants have > 1)	
Family physician, in practice	8 (50)
Health team manager/administrator	3 (19)
Social worker/case manager	3 (19)
Specialist physician	3 (19)
Physician researcher	4 (25)
Registered nurse	2 (13)
Dietitian	1 (6)
Province of practice	
Ontario	10 (63)
Manitoba	3 (19)
New Brunswick	2 (13)
British Columbia	1 (6)
Practice setting	
Large urban	10 (63)
Small urban/rural	6 (38)
Gender	
Female	13
Male	3

Participants had created and implemented a wide range of social interventions including direct team-based services such as embedded income security and legal specialists, social needs data collection and screening, benefits guidance tools for front line health providers and offering clinical services in non-traditional locations. They developed partnerships with community and government agencies to facilitate access to benefits and other support services. They improved access to care for underserved communities, including those living on low income, the precariously housed, new immigrants, and Black and Indigenous people, through outreach programs, accepting referrals from non-traditional sources and increasing flexibility in team structures. Finally, participants were active in health professional organizations and academic health sciences faculties embedding action on social determinants of health (SDOH) and critical exploration of health inequities in training curricula for students, postgraduate trainees and practicing health professionals.

While settings and programs varied, participants’ experiences point to core practical elements they saw as crucial to successfully embedding these programs in routine primary care practice.

### Understanding community needs

Participants highlighted the importance of establishing a detailed understanding of patient and community social needs, both by using data and through direct engagement with potential program users.

Participants used data to delineate communities and to understand who is not seeking care in order to target interventions. As one urban health team manager explained:

*Equity really involves understanding who are the people that live and reside within your catchment area that you’re not currently serving*. *And if they are in need of care*, *why aren’t they accessing care*? *[P05*]

A family physician in a large urban health team pointed to the gaps in knowledge that can be revealed by community-level data:

*We practice in*… *an area where most of our patients are well off*… *we’re not seeing patients*… *who are down and out and really struggling*… *[But] I knew that in our [catchment area]*… *there’s a poverty rate*… *above the provincial average*. *[P04*]

While community-level data was clearly important, participants also discussed the limits of data in revealing certain kinds of marginalization. Here, a family physician educator draws on her lived experience to teach medical learners:

*When I tell them that two-thirds of the people who use our food banks in [province] have a job*… *a lot of the people will say*, *“Oh*, *they live on welfare because they’re lazy*,*” blah*, *blah*, *blah*, *blah*. *… I show them a picture of me when I was a teenager and I tell them*, *“Would you have asked this teenager if she’s struggling at home and she has problems making ends meet*?*” And … they see it’s my face and they’re like*, *“Uh*.*” I say*, *“Yeah*, *it’s a trick question*.*”*… *I was living in poverty*, *we were food insecure*. *As a family we were homeless when I was that age*, *and I say “It’s not written anywhere on my face or on my clothes or in my hair that that’s where we were living*. *So don’t assume anything*.*” [P03*]

### Improving access to care

Participants described ways the traditional structure and approaches of health teams can exclude socially marginalized clients. They saw efforts to reduce barriers to access as a necessary precondition to effective social interventions both because patients without a primary care provider are unable to benefit from primary care-based social interventions and because social needs pose barriers to accessing health team services.

A health team nurse manager described how they have trained staff to recognize and address client needs from the point of first contact:

*We have algorithms in place for our phone centre agents so that when individuals*… *say*, *“I don’t have a postal code*,*” that’s an automatic trigger just to accommodate*. *[P05*]

A community-based pediatrician discussed shifting traditional referral and communications processes to improve access for marginalized children and their families:

*Maybe you start taking referrals from people that aren’t just doctors*. *Like we worked to make sure that Medicare was okay with social development sending us a referral*… *I kind of believe in teachers*, *as well*, *in resource teachers*. *… We do school-based clinics*. *We do school meetings*. *We allow people to email you*, *like a school for feedback*. *So*, *just being a little bit more nimble on how we gather information*. [*P11*]

A health team manager similarly suggested that access to care can be improved by offering services in non-traditional locations:

*We will see patients*, *clients*, *in home visits*, *in places that they designate as safe*. *So*, *we’ve done diabetes education at Tim Hortons*, *we’ve done respiratory education in the mall in our community area*… *We’ve actually moved*… *our health education groups*, *our mental health support groups*, *out of the traditional medical sites*, *into*… *community centres*. *[P08*]

### Building safe care spaces

Many participants noted that client experiences with health institutions that perpetuate inequity or systemic trauma poses a barrier to attendance and engagement with care. A pediatrician spoke of the education she led, focused on health team members’ attitudes toward socially marginalized clients:

*We had a pretty significant no-show rate*… *So*, *I spent a lot of time*… *changing that culture of*, *“Oh they didn’t show up” or “they were non-compliant” and that kind of labelling and assumption*, *[to]*… *saying okay*, *well why didn’t they show up and then trying to work with people to say*… *would they feel more comfortable with a house call or being seen in the school or do we need some sort of buffer of trust*. *Would they want a social worker there or a teacher there or something*? *[P11*]

Similarly, a family physician described an approach to direct patient care that explicitly demonstrates safety from the first point of contact:

*When I see somebody for the first time*, *a lot of what I’m really trying to do is signal safety*, *signal that this is a non-judgemental space*, *that you can come back to see me for whatever it is that you need*… *trying to determine*… *what their priorities are*… *They may be somebody who clearly has a very severe mental illness*, *but they just want to talk to you about the fungal infection on their foot and so*… *maybe noting down some of what you’re seeing and thinking about things you’d like to return to if the person comes back*, *but really*, *otherwise just addressing what they want*. *[P13*]

### The importance of consultation

Participants emphasized the need to engage all partners—health team staff, community members, organizations, and patients–in program development.

A health team manager illustrated her attempts to increase buy-in for home supports for frail seniors, by engaging health agency partners and health team staff in program design. Government-funded coordinators embedded in a health team gained a far deeper understanding of patients’ lived realities, and were able to advocate more effectively for their needs:

*It’s no secret that homecare had a really bad rep for years*… *as very bureaucratic*, *very paper driven*, *and very little hours provided to patients*. *But*… *the people that were working were phenomenal*, *they were very passionate about their work and really wanted to make a difference*. *So*, *it was really important from day one that*… *this is a co-designed approach*… *It wasn’t me and my equivalent at [home care agency] hammering out the details around what was going to happen*, *it was the actual care coordinators that were going to be working with us*, *and the physicians and other members of the team working together around envisioning what this could look like*. *[P06*]

Another manager emphasized the perspectives gained by consulting patients and community members in the development process:

*We brought a group of nine*… *[patients] together to better understand some of the challenges that they face in terms of self-managing their diabetes and to get their input in terms of what we could do to better support their care*… *What they said was … “Yeah*, *I eat like crap*, *but I’m also homeless and the food that they provide me at these shelters is actually starch-based*.*”*… *Or “Yes*, *I’m overweight*, *but I’m actually overweight because I’ve suffered trauma*.*” [P05*]

### The importance of partnerships

Participants demonstrated that successful social intervention programs require broad engagement of both internal team members and external partners and supports. One health team manager, whose program supports solo community physicians, suggested that intervention programs should feel like they extend capacity.

*You would still get*… *providers that say*, *“Hey*, *I can’t cure poverty*.*” But*… *they’re starting to look at their larger team being way beyond their clinic’s walls*… *And one of the things I worked really hard with each clinic around is to say*, *“Your team is bigger than you think”*… *managing a patient panel of 1200 becomes much more reasonable*… *if you realize you’re not doing it alone*. *[P08*]

A family physician discussed the power of repurposing and expanding health team resources, as well as connecting with community agencies:

*Your team can be internal*, *and your team can be external*… *connecting with some of the resources in your community*, *for example*, *your neighbourhood legal clinic or your neighbourhood drop-in*, *figuring out where the nearest housing worker is*, *where the nearest Early Years Centre is*… *we’re often the first stop for people*, *so they actually might not have yet looked into … whatever it is that would be beneficial for them*. *[P13*]

Many participants discussed the challenges to program development posed by longstanding silos between health care and other organizations. An income security focused case worker discussed the importance of inter-agency meetings:

*We meet once a month–and this is with managers from primary care*, *mental health*, *public health*, *housing*, *[health team] representatives*, *adults’ day hospital*, *[province] Housing*… *Social Assistance*, *and we discuss difficult cases*. *So*, *if there’s a client experiencing systemic barriers—and that’s kind of the key point for this is the systemic barrier piece—we try to resolve the issue*. [*P02*]

Several participants had created effective partnerships with government agencies. In one setting, a federal agency attended family-oriented events to issue ID documents:

*One of the barriers for people to access government benefits and file their income tax is not having the proper ID*. *Service Canada issues social insurance numbers*… *so*, *for example*, *we have a preschool milestones clinic coming up*… *there’s approximately 200 to 300 families that attend that and that’s to get their preschoolers ready for school*. *They get hearing screening and dental screening*. *Public Health nurses are there as well*. *We have dieticians doing the nutrition screening*. *And so this year for the first time we are partnering with Service Canada and they’re going to be having a booth there*, *helping families get their social insurance numbers*, *talk to them about getting their children’s birth certificates because they do need a birth certificate to register for school*. *Also*… *we are starting to promote underutilized benefits such as the Canada Learning Bond*. *[P16*]

In another example, a health team manager described bridging the gap between health providers who are traditionally wary of government services and government-funded supports for socially marginalized clients, especially those with high mental health needs. An income security-focused case manager described such a direct partnership with local social assistance offices:

*A significant portion of the clients that I work with are on social assistance*. *And they may have complications if they didn’t provide the proper form*, *or they’re cut off for a specific reason*. *And*, *instead of simply sending an e-mail or a letter to the worker and not finding out for an extended period of time*, *I’ve been able to build that relationship with the different offices that we are able to communicate*, *that we are working together to better help clients and it’s showing very positive results*… *and it goes even further*… *I have*, *once a month for a few hours*, *a worker come down into one of the primary care clinics that’s very underserviced*. *[P02*]

### Developing simple, practical tools

Participants valued simple, practical social intervention tools that can be easily integrated into care pathways to facilitate health provider engagement. One health team manager described one-page summaries of their services that they hand out to potential partners:

*Each one of our clinicians does a one-page*… *service description summary*… *tangible things*. *… if I*… *preface the meeting with*, *“Here’s an email with all these attachments*, *please have a review*, *we can discuss at our meeting*,*” they then can see*, *“Oh*, *I would refer to this person if my*… *patient was having challenges with accessing [government income programs for seniors]” [P08*]

A family physician, similarly, spoke of a simple social prescription form that served as an easy referral to services:

*They’ve developed a prescription sheet that you kind of tick off resources based on resources that patients would need with regards to social determinants of health*… *it was very practical*. *[P15*]

And a nurse with a focus on social determinants of health developed a business card for her program as a simple reference for health provider partners:

*We developed a business card and*… *on the front it says*, *“Do you have trouble making ends meet at the end of the month*?*” And then it has the Get Your Benefits website*… *And then on the back we showcase Government of Canada benefits finder and … a Government of [province] residence portal … just because there’s a lot of things that change with benefits and credits and they’re the experts*. *[P16*]

### Targeting institutional change

Participants looked beyond their immediate clinical spaces, to the importance of normalizing social intervention programs among all health care providers and health system planners. Two family physicians discussed the need for respected local health care providers and health provider leaders to build support for this approach to care.

*I feel like just us coming in to talk about it won’t get the buy-in from physicians*, *but if it’s their peers talking about that*, *that will make a bigger difference in whether physicians take this on*. *[P14*]

*… it’s amazing for me to see someone like [physician]*, *who really has been the leader in social accountability work in family medicine*, *…*. *I think that helps other family doctors say*, *“Oh*, *this is someone who looks like me*, *who understands the environment in which I was trained*, *and yet they’re doing this*. *How can I do it too*?*” [P13*]

## Discussion

The field of social interventions in primary care is growing rapidly, with marked interest from health practitioners, communities, and health funders. Addressing social health in primary care practice poses a complex challenge to health providers and health systems planners. This study offers the learnings of pioneers in this field. Focused on front line experience with program design and implementation, these learnings provide valuable information to those seeking to further expand and normalize programs to address social risks to health.

This study offers the first qualitative exploration of lessons learned from implementing social interventions with a geographically diverse sample of primary care-based experts working in a range of practice settings on the front lines of care. This work complements previous qualitative studies that explored primary care providers’ understanding of inequalities [13] and examined specific social interventions [21]. Other research focused specifically on improving equitable access to care [20], and on the evaluation of social interventions [23]. This study takes a broad approach to understanding the practical experiences of front-line practitioners.

Participants demonstrated an ability to engage with complex social risks and inequities, and to translate their experiences into interventions in front line health care settings. These innovators paired a long-term vision for change with a deep understanding of the practical barriers to the implementation of novel social risk-focused programs. They were able to design and implement these programs by understanding and consulting with their target communities as well as the institutions through which the programs were delivered.

Program designers facilitated change through motivational leadership, education, the use of simple practical tools and the tangible addition of resources to teams through increased internal funding and external partnerships. They paired this front-line innovation with an understanding of the institutional and social structures that underlie and reinforce social barriers to health.

Their experiences point to the creativity and persistence required to shift the culture of health care to take responsibility for under-addressed risks to health. Participants did not question the need for these interventions, and their persistence and commitment is consistent with literature demonstrating an increase in provider wellness when social risks are addressed through front line care [17, 18].

This study’s findings offer perspectives from practitioners that begin to frame commonalities in approach and experience to be tested by future researchers exploring social interventions in primary care. As experience in this field grows, learnings can be consolidated into models of understanding and models of change to be applied to health teams and health systems interested in engaging with social interventions. This study contributes an important set of such learnings, which can be combined with other emerging knowledge, including that in the scientific literature and the perspectives of people with lived experience of social marginalization, to develop systematic understandings of the change processes required to embed social interventions into primary care.

### Limitations

This study focused on Canadian health care innovators in four provinces. Social intervention development is, to some degree, context-specific, and may require different strategies in different provincial or national contexts. As there is not, yet, a simple way to identify leaders in this field, the search for participants depended on professional networks, internet searches and snowball sampling. There may be practitioners working in other contexts (including other provinces and different practice settings) who were not identified through this process. Future research should aim to expand the scope of experience of participants. While participants represented a range of health professionals and managers, the perspective of program users and people with lived experience of the inequities addressed by these programs is necessary for a complete analysis of their utility and relevance. These interviews did not focus on interventions targeted at Indigenous inhabitants of this land as the social risks posed by their particular history in relation to the health care system and by colonial oppression warrants dedicated exploration.

These interviews were conducted prior to the COVID pandemic. The pandemic brought increased attention to inequities in health care access and outcomes. This shift may represent a heightened opportunity for the introduction of programs that directly address such inequities.

## Conclusion

Social interventions are rising in importance and impact in front line health care. This study of leaders in program development and implementation demonstrates a deep commitment to, and excitement about, social interventions. It highlights the need for creativity, persistence, partnership, a deep understanding of community and individual social needs, a willingness to overcome barriers, and an ability to focus on institutional change to successfully develop and implement social intervention programs in primary health care settings.
